# Linear Feature Projection-Based Sensory Event Detection from the Multiunit Activity of Dorsal Root Ganglion Recordings

**DOI:** 10.3390/s18041002

**Published:** 2018-03-28

**Authors:** Sungmin Han, Inchan Youn

**Affiliations:** 1 Biomedical Research Institute, Korea Institute of Science and Technology, 5, Hwarang-ro 14-gil, Seongbuk-gu, Seoul 02791, Korea; han0318@kist.re.kr; 2 Division of Bio-Medical Science &Technology, KIST School, Korea Institute of Science and Technology, 5, Hwarang-ro 14-gil, Seongbuk-gu, Seoul 02791, Korea

**Keywords:** sensory event detection, multiunit activity, linear feature projection, projection pursuit, negentropy maximization, tactile afferent

## Abstract

Afferent signals recorded from the dorsal root ganglion can be used to extract sensory information to provide feedback signals in a functional electrical stimulation (FES) system. The goal of this study was to propose an efficient feature projection method for detecting sensory events from multiunit activity-based feature vectors of tactile afferent activity. Tactile afferent signals were recorded from the L4 dorsal root ganglion using a multichannel microelectrode for three types of sensory events generated by mechanical stimulation on the rat hind paw. The multiunit spikes (MUSs) were extracted as multiunit activity-based feature vectors and projected using a linear feature projection method which consisted of projection pursuit and negentropy maximization (PP/NEM). Finally, a multilayer perceptron classifier was used to detect sensory events. The proposed method showed a detection accuracy superior to those of other linear and nonlinear feature projection methods and all processes were completed within real-time constraints. Results suggest that the proposed method could be useful to detect sensory events in real time. We have demonstrated the methodology for an efficient feature projection method to detect real-time sensory events from the multiunit activity of dorsal root ganglion recordings. The proposed method could be applied to provide real-time sensory feedback signals in closed-loop FES systems.

## 1. Introduction

In functional electrical stimulation (FES) systems, feedback signal-based closed-loop control is a useful approach to restore lost motor functions. This allows fine and highly complex motor functions to be controlled based on sensory feedback signals [1,2,3]. The use of a neural electrode directly connected to the nervous system may provide an abundance of natural sensory information and many studies have been conducted to provide feedback signals using neural activity recorded from the nervous system. For example, afferent signals, such as tactile and proprioceptive activities, have been used to extract sensory information for discriminating sensory events [4,5] and decoding limb movements [6,7,8]. These studies have focused on extracting the most informative feature vectors and have proposed sophisticated decoding algorithms for feedback control.

Recently, multiunit activity has received considerable attention in the analysis of extracellular recorded neural signals. Multiunit activity refers to simultaneous summation recordings from a combination of multiple neuronal spikes surrounding the electrode channel that describe the local neuronal network dynamics [9]. Unlike single-unit activity, identification of individual neuronal units is not necessary in multiunit activity; accordingly, signal processing is simple and rapid. In contrast, the process of spike sorting is often time consuming and typically requires human operators [10]. Thus, several studies have used multiunit activity to extract information from neural activity. For example, multiunit activity, such as the root mean square of multiple spikes recorded from intracortical activity [11], the unsorted threshold-crossing events recorded from the primary motor cortex [12], and the mean absolute value of the afferent signals in the dorsal root ganglion [13], has been used to predict limb movements. However, the use of multiunit activity is intrinsically insufficient to provide relevant information about the neural activity relative to single-unit activity; this activity reflects the aggregate spiking activity regardless of the number of neurons and cannot represent the spike information corresponding to the various neurons. This inherent deficiency reduces the likelihood of achieving high decoding performance when multiunit activity is used as a feature vector to extract neural activity information.

To address these considerations, the current study investigated an efficient feature projection method to improve the detection accuracy of sensory events from the multiunit activity of tactile afferent signals. Generally, feature projection can reduce the dimensionality of the feature vector and decrease the learning parameter of a classifier. Additionally, such projection produces a well-described coordinate system for the distribution of features. As a result, classifiers can find a decision surface with an enhanced separation margin from the feature space with improved class separability, thereby improving the classification accuracy and speed [14]. Principal component analysis (PCA) has been widely used as a linear feature projection method [5,13,15,16]. PCA preserves maximum variance while transforming the original feature vector into a small number of uncorrelated coordinates to reduce the dimensionality of the feature vector without loss of the original information. However, the distribution of different classes is not always precisely separated in the PCA-projected feature space because PCA produces a distribution of all features based on the maximization of the projection variance irrespective of the class separation. When sensory events were generated by mechanical stimulations on different areas of the rat hind paw, cutaneous afferents propagated independently along the individual fascicles of both the femoral and sciatic nerves; their soma were gathered in the L4 dorsal root ganglion [17]. Consequently, neural signals recorded from the L4 dorsal root ganglion using the multichannel microelectrode can be expected to contain mixed neuronal signals of independent afferent activities and to contain diverse temporal patterns which are dependent on the various sensory events. A linear combination of independent source signals can be estimated from linearly mixed recordings by the independent component analysis (ICA) method. Additionally, individual fascicular signals from peripheral nerve recordings have been successfully separated by the ICA method to provide useable information [18].

In this study, a linear feature projection method composed of the projection pursuit and negentropy maximization (PP/NEM) was used to improve the detection accuracy of sensory events. PP/NEM is intended for ICA and is suitable for performing source separation from linearly mixed recordings. PP/NEM measures the negentropy of estimated signals which is equivalent to the degree of mutual independence. Maximum mutual independence is achieved by maximizing the negentropy of the estimated signals. Therefore, independent source signals can be extracted by maximizing the negentropy from the mixed recordings. Tactile afferent signals were recorded from the L4 dorsal root ganglion using a 16-channel microelectrode during sensory events for different stimulus locations on the rat hind paw; these produced multiunit spikes (MUSs) in each electrode channel which were extracted as multiunit activity-based feature vectors. PP/NEM was used to project the feature vector and a multilayer perceptron (MLP) was used as the classifier to discriminate the various sensory events. The detection accuracy of the proposed method was compared to that of other linear or nonlinear feature projection methods.

## 2. Materials and Methods

### 2.1. Signal Processing Procedure

**Figure 1:**
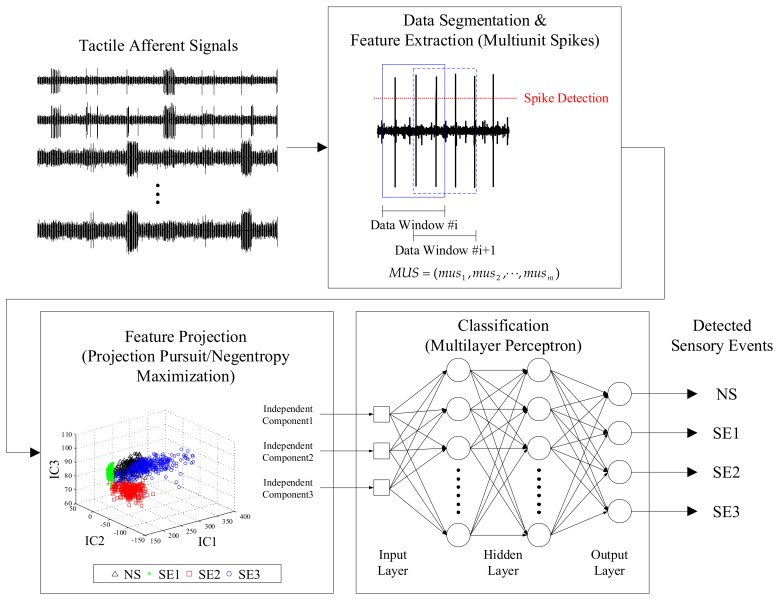
A block diagram of the proposed projection pursuit and negentropy maximization-based sensory event detection method using multiunit activity-feature vectors from tactile afferent signals. NS, SE1, SE2, and SE3 represent non-stimulation, sensory event 1, sensory event 2, and sensory event 3, respectively.

Figure 1 shows a block diagram of the proposed PP/NEM-based sensory event detection method using the multiunit activity-feature vector from tactile afferent signals. Neural signals were recorded from the L4 dorsal root ganglion using a 16-channel microelectrode. The signals were then segmented into a data window and multiunit activity-based feature vectors were extracted based on the MUSs from each segmented signal. MUSs were projected using PP/NEM, thus reducing the dimensionality. An MLP classifier determined the learning parameters using PP/NEM-projected feature vectors and the maximum output was selected as each decision of the sensory event.

### 2.2. Sensory Event Application and Tactile Afferent Signal Recording

The method of sensory event application and tactile afferent signal recording was based on a previous study with some modification [5]. All animal experimental procedures were approved by the Institutional Animal Care and Use Committee of the Korea Institute of Science and Technology (Certificate number: 2017-019). The experimental protocol was performed in accordance with the recommendations for the care and use of laboratory animals. Five adult male Sprague-Dawley rats weighing 250–350 g were used in the experiments. The rats were anaesthetized using an intraperitoneal injection of sodium pentobarbital (40 mg/kg), and anaesthesia was maintained by administering additional doses (20 mg/kg) as needed. When sensory events are generated by mechanical stimulation, tactile afferent activities propagate along the afferent fibres of the sensory neurons. The afferent fibres of the saphenous nerve project to the femoral nerve plexus of L2, L3, and L4 dorsal root ganglia, while those of the tibial and sural nerves project to the sciatic nerve plexus of L4, L5, and L6 dorsal root ganglia [19]. Femoral and sciatic afferent neurons are shared only in the L4 dorsal root ganglion and topographical distributions of their neurons differ. While femoral nerve neurons are distributed in the dorsal and rostral regions of the L4 dorsal root ganglion, the sciatic nerve neurons are distributed in the medial and ventral regions [17]. Consequently, the activity of both the femoral and sciatic nerve neurons can be obtained simultaneously in the L4 dorsal root ganglion using multichannel microelectrode recordings.

**Figure 2:**
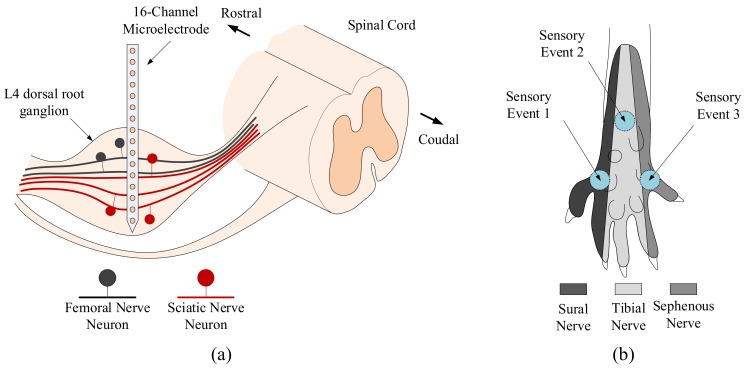
The scheme for tactile afferent signal recording and sensory events. (**a**) Simplified illustration for the femoral and sciatic nerve neurons distribution of the L4 dorsal root ganglion. Tactile afferent signals were recorded from the L4 dorsal root ganglion using a microelectrode in which the neurons of the femoral and sciatic nerves are shared in the L4 ganglion. (**b**) Sensory events were generated by mechanical stimulation of three different areas of the rat hind paw in accordance with the cutaneous receptive fields of the three afferent nerves: the sural, tibial, and saphenous nerves.

Considering the neuroanatomic pathway of the afferent nerves, neural signals were recorded from the L4 dorsal root ganglion. The rat was positioned on a stereotaxic apparatus (David Kopf Instruments, Tujunga, CA, USA) using a mouthpiece, ear bars, and vertebrae clamps. The L4 dorsal root ganglion was exposed by laminectomy and a 16-channel microelectrode (A1x16-5 mm-50-703-CM16, NeuroNexus Technologies, Ann Arbor, MI, USA) was inserted perpendicularly into the left L4 ganglion (Figure 2a). Each electrode was arranged on a 1 × 16 layout of 16 iridium channels with a 30 µm diameter and 50 µm interchannel distances on a 5 mm long single-shank silicon substrate. Neural signals were digitized at a 32 kHz sampling rate and bandpass filtered between 300 and 5000 Hz using a digital data acquisition system (Neuralynx, Tucson, AZ, USA). Reference and ground wires were placed subcutaneously in the back of the rat.

Sensory events were generated by mechanical stimulation to elicit the responses of three different afferent nerves. Stimulation areas were selected based on the cutaneous receptive fields of the nerves; the left, middle, and right third of the plantar surface of the rat hind paw are innervated by the sural nerve, tibial nerve, and saphenous nerve, respectively [19]. The stimulations were applied to three different areas of the plantar surface of the rat hind paw using a von Frey monofilament (North Coast Medical Inc., Morgan Hill, CA, USA) with 10 g of bending force. Therefore, sensory events comprised three parts: sensory event 1 (SE1), sensory event 2 (SE2), and sensory event 3 (SE3). SE1, SE2, and SE3 were targeted to the areas identified by the sural nerve, tibial nerve, and saphenous nerve, respectively (Figure 2b). Each session of sensory events was composed of alternating stimulation and non-stimulation (NS) periods and sequentially occurred 15 times. The periods of stimulation and NS were identically set to approximately 1.5 s and synchronized with a metronome; the duration of each session was approximately 45 s. Neural signals were collected from five rats and ten sessions were conducted for each rat. The data acquisition procedures have been described in more detail in a previous study [5].

### 2.3. Data Segmentation

Corrective motor commands can be generated based on real-time sensory feedback signals and the maximum acceptable delay time in the sensorimotor control system can be regarded as approximately 300 ms [20,21]. For a series of dexterous manipulation tasks, the required motor actions associated with the sensory events should be accurately predicted without time delay. In this study, a moving window scheme was used to detect sensory events for real-time implementation. This approach allowed the steady-state signal to be analysed in real time to produce a continuous decision output [20]. Two parameters were selected to implement a sliding window technique: a data window and a window increment. The data window was segmented from the acquisition data into multiple frames and feature extraction was performed on each data window. The window increment was determined by the setting of the overlap length and provided the processing time of the classifier. Considering the maximum acceptable delay time, the data window and window increment were set to 200 ms (6400 samples) and 100 ms (3200 samples), respectively, so that sensory events could be detected within 200 ms [5].

### 2.4. Feature Extraction

Signals from individual neurons generally represent the neural activities and single-unit activity-based feature vectors that are often used to provide information results regarding higher-order functions of the sensorimotor system [22]. Activities of multiple neurons are simultaneously detected in the electrode channel from extracellular recordings; thus, the process of sorting is necessary to discriminate the activity of individual neurons from the multiple neurons. Spike sorting presents limitations in terms of processing efficiency and reliability [10,22]. Additionally, high computational costs and complex algorithms are required to achieve a high degree of spike-sorting accuracy. These situations are problematic for real-time applications. Thus, we used multiunit activity-based feature vectors to represent the neural activity information.

To extract the multiunit activity-based feature vector, MUSs were constructed from the segmented tactile afferent signals. Each channel of the electrode was treated as a single putative neuron and the threshold was set to the mean of the baseline noise plus five times the standard deviation of the mean value for each channel [5]. Each spike was composed of 50 sample points corresponding to a spike duration of 1.6 ms. The refractory period was set to 0.5 ms to prevent double counting of the same spike. The number of multiple spikes detected by the threshold crossing events was calculated as the MUS value in each 200 ms bin corresponding to the data window period for each channel. The MUS is defined as

(1) $$MUS=(mus_{1},mus_{2},\cdot \cdot \cdot ,mus_{m})$$

where $mus_{m}$ is the number of spikes of the m-th channel in each bin. Therefore, the m-dimensional feature vector was generated as the MUS.

### 2.5. Feature Projection

The dimensionality of the feature vector can be reduced by the feature projection method. This produces the low-dimensional feature vector through a transformation from the original feature space into a subspace, thereby decreasing the learning errors of the classifier and increasing the classification accuracy. Three different feature projection methods were investigated to achieve a high detection accuracy of the sensory events. PP/NEM and PCA were used as linear feature projection methods, whereas a self-organized feature map (SOFM) was used as a nonlinear feature projection method.

Projection pursuit (PP) is the process of estimating a matrix by optimizing a project index to find the directions of the most interesting low-dimensional subspace from the high-dimensional data sets. PP can be expressed as

(2) $$y=W^{T}x$$

where x is the m-dimensional original feature vector, y is the n-dimensional projected feature vector (m≥n), and W is the m×n matrix. PP is closely related to ICA which can be used to obtain the direction by minimizing the mutual information between the projected data. Minimizing the mutual information of the projected data is equivalent to finding directions that maximize negentropy. Therefore, an invertible transformation W can be found by negentropy maximization [23]. Negentropy, J(y), is defined as

(3) $$J(y)=H(y_{G})-H(y)$$

where H(y) is the entropy of a Gaussian random vector y and $H(y_{G})$ is a Gaussian random vector with the same covariance matrix as y. The value of W can be found by maximizing J(y), which was implemented based on the PP/NEM method [23,24]. For computational simplicity, the original data x is centred to generate the zero mean and whitened to set the unit variance. A new vector $\tilde{x}$ is obtained by using the eigenvalue decomposition of the covariance matrix $E\{xx^{T}\}=EDE^{T}$, where E is the orthogonal matrix of the eigenvectors of $E\{xx^{T}\}$ and D is the diagonal matrix of the eigenvalues of $E\{xx^{T}\}$. One projection direction, $y_{i}=w^{T}x$, can be obtained by maximizing the function $J_{G}(w)$ given by

(4) $$J_{G}(w)={\left[E\{G(w^{T}x)\}-E\{G(v)\}\right]}^{2}$$

where G is a non-quadratic function and v is a Gaussian variable of the zero mean and unit variance. The optimum projection direction can be calculated as

(5) $$w^{+}=E\{xg(w^{T}x)\}-E\{g^{\prime }(w^{T}x)\}w\;w^{new}=w^{+}/\left\|w^{+}\right\|$$

where g is approximated given that $g_{1}(y)=\tanh(a_{1}y)$, $g_{2}(y)=y\exp(-a_{2}y^{2}/2)$, or $g_{3}(y)=y^{3}$ are suitable choices for a contrast function and $1\leq a_{1}\leq 2$ and $a_{2}\approx 1$ are constants. The equation is iterated until the direction of the optimal projection is found. To find more than one direction of projection, a newly found direction of projection is estimated individually on the basis of the Gram-Schmidt orthogonalization which is performed for every iteration as follows:

(6) $$w_{n+1}^{+}=w_{n+1}-\sum_{j=1}^{n}(w_{n+1}^{T}w_{j})w_{j},\;j=1,\ldots ,n\;w_{n+1}^{new}=w_{n+1}^{+}/\left\|w_{n+1}^{+}\right\|$$

This approach estimates the projection directions one by one. We have already estimated n vectors $w_{1},\ldots ,w_{n}$, and the projections of previously estimated n vectors are subtracted every iteration step from $w_{n+1}$. In this study, $g(y)=y\exp(-a_{2}y^{2}/2)$ was used as a contrast function, and a three-PP direction was extracted as a dimensionality-reduced feature vector considering the sensory events of three different areas related to three different afferent nerves. Therefore, the m-dimensional feature vector was projected onto the three-dimensional subspace.

PCA is a linear orthogonal transformation that converts a set of correlated observed variables into a new set of uncorrelated variables by maximizing the variance along the axis of the principal components. The process of linear projection is expressed as $y=W^{T}x$, where x is correlated to the observed variables of the m-dimensional original feature vector and y refers to the uncorrelated variables of the n-dimensional projected feature vector. First, an m×m covariance matrix was constructed from the original feature vectors and the eigenvalues and eigenvectors were calculated from the m×m covariance matrix. Then, the n eigenvectors corresponding to the n largest eigenvalues were selected to set the rows of the W matrix. Considering the sensory events of three different areas related to three different afferent nerves, the first three principal components were used to reduce the dimensionality; accordingly, the m-dimensional feature vector was projected onto the three-dimensional subspace.

The SOFM nonlinearly transforms the original feature vector into a new feature space by mapping onto a two-dimensional (2-D) lattice of neurons, where each neuron is a weight vector fully connected to the input data [25]. The synaptic weight vectors are adjusted on the basis of their similarity to the input data and the topological neighbourhood of the winning neuron. The synaptic weight vectors of all the neurons are adjusted using an updated rule:

(7) $$w_{k}^{new}=w_{k}^{old}+\eta (x-w_{k}^{old})$$

where x is the input data of the m-dimensional original feature vector, $w_{k}$ is the k-th weight vector, and η is the learning rate. The winning neuron i(x) is determined by the Euclidean distance:

(8) $$i(x)=\underset{k}{\arg\min}\left\|x-w_{k}\right\|,\;k=1,\ldots ,l$$

where l is the number of lattice sites. The winning neuron is found by identifying the best similarity between its weight vector and the input feature. The input layer of the SOFM was composed from the m-dimensional original feature vector. In consideration of the processing time and feature map resolution, the output layer was set to a 40 × 40 2-D lattice. Therefore, the m-dimensional feature vector was mapped into a node in the 2-D lattice.

### 2.6. Classification

After the feature projection, an MLP was used to detect the sensory events. The MLP is a feed-forward artificial neural network that is useful for solving nonlinear classification problems. The MLP consists of three layers: an input layer, a hidden layer, and an output layer. Each node of a layer was fully connected to the other nodes of the layer and a bipolar sigmoid was used as the nonlinear activation function. The input layer was constructed from the outputs of the feature projection. The number of hidden layers was set to two and each hidden layer had ten neurons. The output layer consisted of four neurons for the three types of sensory events with NS. The learning process was repeated until the mean squared error was sufficiently small and set to 0.01 for the stopping condition. The maximum output was used to detect the sensory events from the MLP output. The network structure was optimized through a trial-and-error procedure and the connecting weights and biases were adjusted using an error backpropagation algorithm.

### 2.7. Performance Evaluation

The performance of the proposed method was evaluated with respect to detecting the sensory events from the tactile afferent activities recorded from the L4 dorsal root ganglion using a 16-channel microelectrode. Sensory events were induced by mechanical stimulation of three different areas, each of which is related to a different afferent nerve of the plantar surface of the rat hind paw. The recorded tactile afferent signals were segmented to a 200 mm data window and MUSs were extracted from each data window as a multiunit activity-based feature vector. Feature projection was implemented using PP/NEM and the projected feature was subsequently used as an input for the MLP classifier. Each decision was selected as a sensory event according to the maximum output. The proposed method was conducted using an Intel^®^ Core™ i3-4100M 2.5 GHz PC with 4 GB of RAM.

The total datasets were collected from each of the five rats and the classification performance was evaluated using 10-fold cross-validation. The datasets were randomly divided into ten equal subsets in which nine subsets were used as learning data and one subset was used as test data. The PP/NEM and MLP parameters were determined using the learning data and the detection accuracy was calculated as a percentage of the total correct decisions from the test data. The total detection accuracy was computed as the mean of the accuracy in the five rats. All data are presented as the mean and standard deviation (mean ± SD).

To select an optimal feature projection method for detecting sensory events, the detection accuracy was investigated for the various feature projection methods: PP/NEM, PCA, and the SOFM. The dimensionality of the feature vectors was reduced by the feature projection method and was used as an input for the MLP. Accordingly, three-dimensional reduced-feature vectors were constructed from the outputs of PP/NEM and PCA, while 2-D reduced-feature vectors were constructed from the outputs of the SOFM. The output layer of the MLP contained four neurons for the three types of sensory events with NS and the detection accuracy was compared among PP/NEM, PCA, and the SOFM. All statistical analyses were performed using SPSS 15.0 (SPSS Science, Chicago, IL, USA). A two-way analysis of variance (ANOVA) was used to assess the statistical significance of the differences and to remove the effect of the difference among the rats. A confidence level of 95% (*p* < 0.05) was considered to indicate a significant difference and Tukey’s multiple comparison was used for the post hoc analysis.

To evaluate the robustness of the proposed method for sensory event detection, the detection accuracy was compared among the different sensory events in each rat. One-way ANOVA was used to assess the statistical significance of the differences; a confidence level of 95% (*p* < 0.05) was considered to indicate a significant difference, and Tukey’s multiple comparison was used for the post hoc analysis.

## 3. Results and Discussion

### 3.1. Properties of Recorded Tactile Afferent Signals

Manipulation tasks can be characterized as a sequence of action phases separated by contact events [26]. The sensory information of the mechanical contact states is necessary to plan and control object manipulations while tactile afferents convey the physical properties of the object in contact with the hand. Four functionally distinct types of tactile afferents innervate the glabrous skin and the endings of the afferents terminate at different types of organs. Fast-adapting type-I (FA-I) afferents are sensitive to dynamic skin deformation of a relatively high frequency, while slow-adapting type-I (SA-I) afferents are sensitive to low-frequency dynamic skin deformation. FA-I and SA-I afferents represent the contact and release responses, such as contact timing, contact site, direction of contact force, and breaking contact. Fast-adapting type-II (FA-II) afferents are easily excited by mechanical transients and high-frequency vibrations, whereas slow-adapting type-II (SA-II) afferents exhibit sensitivity to static force. FA-II and SA-II afferents reflect transient mechanical events, such as contact or the breaking of contact with objects and weight information [26].

**Figure 3:**
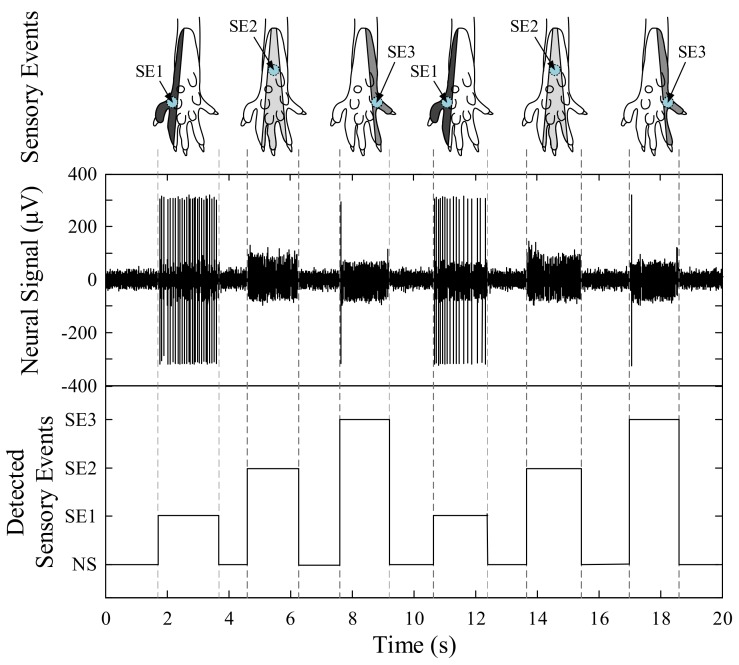
Typical examples of recorded tactile afferent signals during sensory events. The top plot shows three types of sensory events. SE1, SE2, and SE3 represent sensory event 1, sensory event 2, and sensory event 3, respectively. The second plot shows representative neural signals from one channel. Different temporal activities were observed for the different sensory events, and the signals were mixed fast-adapting and slow-adapting afferents. The bottom plot shows the desired output of the proposed sensory event detection method.

Figure 3 shows a typical example of recorded neural signals from the L4 dorsal root ganglion during mechanical stimulation on the three different areas of the plantar surface of the rat hind paw using a von Frey monofilament. The top plot represents the application of three types of sensory events: SE1, SE2, and SE3. The middle plot is one representative channel of the recorded tactile afferent signal for sensory events. The bottom plot is the desired sensory event detection. The L4 dorsal root ganglion contains neurons of both the sciatic and femoral nerves. These include the sural, tibial, and saphenous afferent fibres which arise from different receptive fields of the rat hind paw and afferent activities are transmitted along the individual fascicles of the sensory neurons. The signal amplitude is associated with the relative distance and orientation between the neurons and the channels of the electrode. Therefore, different spatial patterns of neural activities corresponding to sensory events can be observed in the different electrode channels with each channel having different temporal patterns of neural activities corresponding to sensory events. These patterns of tactile afferent activity are repeatedly elicited by and used to detect sensory events.

### 3.2. Comparison of Various Feature Projection Methods

The detection accuracy of PP/NEM was compared to those of PCA and the SOFM. Table 1 shows the number of recorded channels for each rat. The afferent signals were recorded using a 16-channel microelectrode during the sensory events and six to eight channels of the electrode were simultaneously activated for each of the five rats. Feature vectors were extracted using the MUS and the dimension of the MUS feature was subsequently reduced to three through the PP/NEM and PCA and reduced to two through the SOFM. Table 2 shows the detection accuracy of the various feature projection methods. The detection accuracy of the PP/NEM was approximately 3.24% (*p* < 0.01) and 6.13% (*p* < 0.01) better than that of PCA and the SOFM, respectively, representing a significant improvement in detection accuracy. These results demonstrate that the PP/NEM produced superior performance for sensory event detection from the tactile afferent signals.

Table 3 shows the detection accuracy of the proposed method for the different sensory events in each rat and there were no significant differences among the sensory events (*p* > 0.05). This result indicates that the proposed method is robust in the detection of sensory events.

PCA is the most notable unsupervised dimensionality reduction method. Dimensionality-reduced feature vectors by PCA can approximate the distribution of the original features. However, clusters for the different classes are not exactly separated in the reduced feature space because an original feature space is mapped to an appropriate subspace through the maximization of the projection variance of the original features irrespective of the class separation. The SOFM is an unsupervised nonlinear feature projection method that performs nonlinear mapping of the high-dimensional original feature space into 2-D lattices with enhanced class separation. However, synaptic weight vectors are adjusted because of their similarity to the topology of input data without class information. Therefore, the input data for the various classes are sometimes transformed into the same cloud causing low-classification accuracy [27]. However, PP/NEM is intended for ICA which can estimate independent source signals from the linearly mixed recordings. The L4 dorsal root ganglion includes afferent neurons of the femoral and sciatic nerves; the fibres of the nerve independently transmit afferent responses induced by contact events from the cutaneous receptors. Consequently, the recorded neural signals from the L4 dorsal root ganglion using multichannel microelectrodes can be expected to constitute linear mixed signals of independent neuronal activities. With these signal properties, PP/NEM can represent more relevant information from the recorded tactile afferent signals, thereby achieving higher detection accuracy than PCA and the SOFM.

### 3.3. Performance Evaluation of the Proposed Method

**Figure 4:**
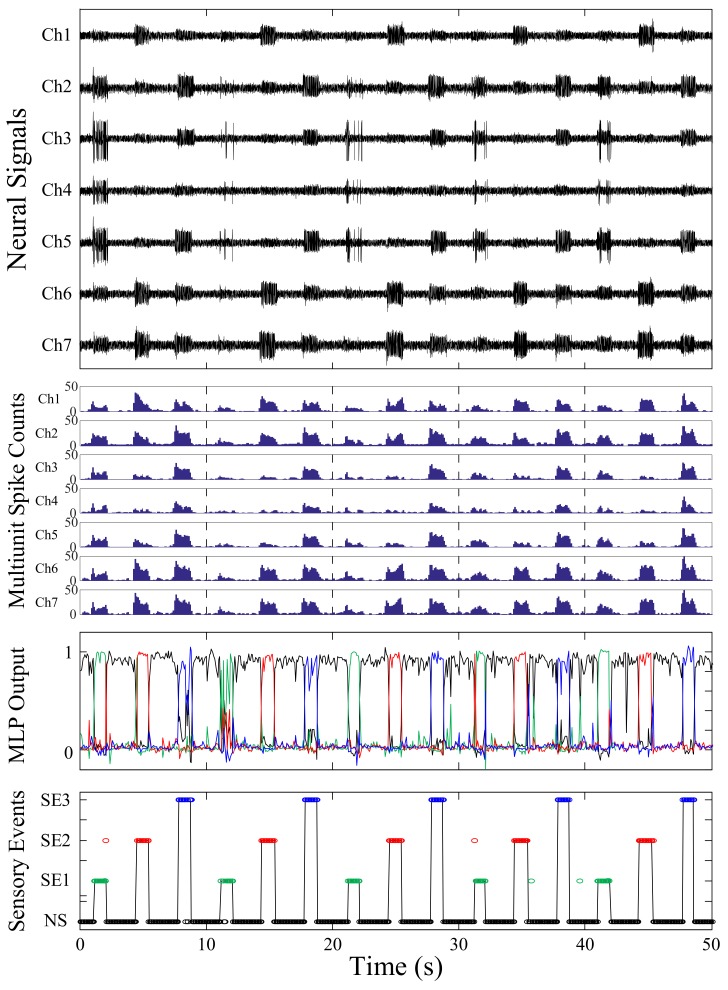
A typical example of the sensory event detection results using the proposed method. The top plot is the recorded tactile afferent signals. The second plot is the multiunit spike counts. The third plot shows the multilayer perceptron (MLP) output values varying between 0 and 1. The bottom plot represents recognition results. The solid line denotes the desired output of the classifier while the black, green, red, and blue open circles denote the sensory event detection results of NS, SE1, SE2, and SE3, respectively.

Figure 4 shows a typical example of sensory events detected using the proposed method. The top plot shows the recorded neural signals from the L4 dorsal root ganglion for various sensory events. The second plot shows the multiunit spike counts of the recording channels. The third plot shows the MLP output values that vary between 0 and 1 in accordance with the tactile afferent signals; the black, green, red, and blue lines correspond to NS, SE1, SE2, and SE3, respectively. The bottom plot shows the sensory event detection results and the maximum values of the MLP output were selected as the sensory events in every decision. The black solid line denotes the desired output and the open circles represent the detected sensory events. The total mean detection accuracy of the proposed method for the five rats was 92.51 ± 2.04%, which was superior to that of the other feature projection methods.

Table 4 lists the total processing time of the proposed method for detecting sensory events. To implement sensory event detection without time-delay, all processes, including feature extraction, feature projection, and classification, should be completed within 200 ms in our data segmentation scheme. The detection output was determined within 10 ms. This result indicates that the proposed method satisfies the real-time constraints and could be suitable for real-time sensory event detection.

Sensory feedback plays an important role in natural motor control and many studies have investigated methods to extract sensory information for providing feedback signals to allow for closed-loop control in FES systems. For example, different types of afferent stimuli were identified from sciatic nerve recordings and classification performance achieved the above median values of 90%, 85%, and 70% for mechanical, proprioceptive, and nociceptive stimuli, respectively [4]. In other studies, the sensory information of limb movement was extracted from dorsal root ganglion recording and used to decode limb positions [6,7]. The decoding accuracy varied depending on the experimental conditions. In this study, sensory events were accurately detected by the proposed method in which above 90% detection accuracy was achieved for each of the five rats. In addition, detection accuracy showed no significant differences among the sensory events in each rat. On the basis of these results, we believe that the proposed method might be a useful approach in detecting sensory events to implement closed-loop FES systems.

Multiunit activity has advantages for typical long-term recordings: neural information can be provided more easily and reliably by multiunit activity over longer recording periods than single-unit activity [11]. Therefore, many studies have considered practical approaches to extracting the neural activities related to motor and sensory information [5,11,13,28,29]. This study used MUSs as feature vectors representing neural information, which were extracted based on multiunit activity and showed satisfactory performance in the detection of sensory events. However, we assumed that relatively simple sensory events were generated by the mechanical stimulation of three different areas of the hind paw and the performance of the proposed method was guaranteed for discrete sensory events. When neural signals were recorded during a real manipulation task, the recorded afferent signals included not only tactile afferents but also proprioceptive afferents. Proprioceptive afferent signals provided sensory feedback of body movements as kinaesthesia information and the relationship between the proprioceptive afferent signals and body movements was dynamic and nonlinear [30,31]. Consequently, further study is necessary to detect various sensory events from afferent signals, including the tactile and proprioceptive activities generated by real movements, to extend the method to describe dynamic relationships.

When a neural signal is recorded from the nervous system using a neural electrode, consideration of long-term stability is important to move this technique towards practical applications. Generally, electrode viability is gradually reduced by deleterious reactive tissue responses such as foreign-body responses and inflammation; additionally, background noise increases over time. Decoding performance would be decreased under low SNR conditions; however, the PP/NEM method has proven to be more capable of extracting discriminative features under high background noise than other conventional methods [24]. In further studies, the decoding performance of the proposed method will be investigated, including the effect of long-term stability.

## 4. Conclusions

We have proposed a method for linear feature projection-based sensory event detection from the multiunit activity of tactile afferents to provide sensory feedback signals in FES systems. The neural signals were recorded from the L4 dorsal root ganglion, a location with simultaneous femoral and sciatic neuron activities. Three types of sensory events were generated by mechanical stimulation on the rat hind paw, considered to be the cutaneous receptive field of three different afferent nerves. The MUS feature was extracted from each segmented signal as a multiunit activity-based feature vector. MUS features were projected using PP/NEM and applied to the MLP classifier to detect the sensory events. The proposed method achieved better detection accuracy than other feature projection methods. In addition, all processes were completed within 200 ms which was sufficient to meet the real-time constraints. These results suggest that the proposed method could be applicable for real-time sensory event detection to provide sensory feedback signals in the closed-loop control of FES systems.

## Figures and Tables

**Table 1:** Number of recording channels for each rat.

Rat	Number of Recording Channels
A	6
B	7
C	8
D	7
E	8

**Table 2:** Detection accuracy for the different feature projection methods.

Rat	PP/NEM	PCA	SOFM
A	91.15	89.41	85.17
B	90.58	88.06	86.10
C	95.75	92.17	89.91
D	92.00	85.94	83.33
E	93.08	90.75	87.41
Mean ± SD	92.51 ± 2.04	89.27 ± 2.41	86.38 ± 2.47

**Table 3:** Detection accuracy of the proposed method for the different sensory events.

Sensory Events	A	B	C	D	E
SE1	90.83	90.32	95.83	91.61	93.33
SE2	91.08	90.13	95.77	92.13	92.64
SE3	90.75	90.26	94.73	92.05	93.03
NS	91.92	91.62	96.67	92.21	93.33
Mean ± SD			92.51 ± 2.04		

**Table 4:** Processing times for the proposed method.

Processes	Processing Time (ms)
MUS	2.73
PP/NEM	1.30
MLP	5.12
Total	9.15
